# On the Uses of Sugar and Lactic Acid in the Animal Economy

**Published:** 1865-05

**Authors:** Samuel Jackson

**Affiliations:** Emeritus Professor of the Institutes of Medicine in the University of Pennsylvania


### SELECTED.

### ON THE USES OF SUGAR AND LACTIC ACID IN THE ANIMAL ECONOMY.

By SAMUEL JACKSON, M. D.,
*Emeritus Professor of the Institutes of Medicine in the University of Pennsylvania.*

The chemical history of the sugars has been very thoroughly worked out by the researches of the chemists. As much can not be said for its physiological actions and uses in the animal organism, or its relations with vital phenomena. On these subjects much valuable information has been obtained from the investigations of Liebig, Lehmann, and Cl. Bernard. They have not, however, so completely exhaused the facts as to render further observations unnecessary, or to cause additional suggestions to be thought obtrusive. With this view, it has appeared to me that a short review of this subject would not be inappropriate.

A brief summary of the principal chemical facts will be required in order to obtain clear ideas of the actions and uses of these bodies in animal organisms. There are several varieties of sugar marked by special characters. Chemists divide them into two kinds or species. The first division or species comprises cane sugar, beet sugar, palm sugar (produced and consumed in India), and maple sugar, with some others of less importance. They are named cane sugars, from their possessing similar chemical properties; and because the Chinese or sugar cane was the plant from which sugar was first made in China, anterior to our historical era, and at the present day nearly 90 per cent. of the sugars of commerce is furnished by various sugar canes.

The second kind or species of sugar consists of glucose or fruit sugar, which exists largely in sweet grapes, and in raisins or dried grapes, from which it has received the name of grape sugar. It is not peculiar to the grape, but is present in nearly all sweet-tasting fruit. The sugar of honey is identical with glucose, which is also the kind of sugar found in the liver, blood, and alimentary canal of animals, and in the muscles, lungs, amniotic and allantoid fluids of the fœtus in its early stages.

The chemical reactions of the two kinds or species of sugar are in strong contrast. The cane sugars are not decomposed by pure potassa and soda; while they are transformed into sugar of the second species, or glucose, by dilute acids and heat. On the contrary, the alkalies potassa and soda decompose glucose, forming peculiar brown-colored acids; according to M. Peligot, the melassic acid. Trommer ascertained that a solution of glucose to which sulphate of potassa and copper were added and heat applied, decomposed the deutoxide of copper of the sulphate by robbing it of one equivalent of oxygen, the protoxide thus formed, being insoluble, is precipitated. This is Trommer's test for diabetic sugar in the urine. Modifications of this test have been made; that of Bareswil is convenient and reliable. It has been adopted by M. Cl. Bernard, who has named it the cupro-potassic test for sugar. M. Becquerel ascertained by experiment that the cane sugars have no actions on the deutoxide of copper, which is not reduced by them.

All the sugars proceed from the transformation of starch. The amylaceous and the saccharine groups of natural bodies are closely allied. They are nearly identical in chemical constitution, consisting of fixed equivalents of carbon (12), with slightly varying equivalents of oxygen and hydrogen in the proportions forming water—that is, from 10 starch, 11 cane sugar, 14 glucose or fruit sugar; or, in other words, they are compounds of a definite proportion of carbon with definite proportions of water. (*Payen.*) Hence their appropriate chemical name carbo-hydrates. From this sameness of composition they are readily transformed into one another. Almost any organic matter in a state of change is capable of effecting this transformation of starch.

In the seeds of the cerealia and of maize an immense store of starch is annually laid up for man and animals. Close in contact with the germ in the seed, imbedded in starch, is placed a small albuminoid substance called diastase. As long as the seeds are kept perfectly dry, even for lengthened periods of time, no action takes place; but as soon as they are exposed to moisture and heat, the diastase exerts a catalytic or fermentative action, and converts the starch into dextrine and glucose. Being very soluble, they immediately become the organizable substance from which is formed vegetable structure. They are promptly absorbed by the germ, and under its organizing force they are transformed into cellulose, the primary organizable material from which are constructed the primary cells and vessels, and the vegetable tissues and organs.

Mialhe has shown that a similar albuminoid (nitrogenized matter) exists in the mixed saliva. Its catalytic power is so great that one part will transform two thousand of starch into glucose. Some physiologists and chemists (Bernard, Robin and Verdeil) do not admit the existence of diastase as a special organic body. They regard it as an albuminoid or nitrogenized substance, in which a change or molecular action is set up by moisture and heat. Its action, they assert, is the same as that of other animal matter in a state of change or starch. The difference, however, is in time. The action of the mixed saliva is most prompt on prepared starch. When a teaspoonful of hydrated starch is held in a sound mouth with no defective teeth, and well washed, to remove any remains of food from between the teeth, in a few seconds it becomes very fluid (dextrin), in a few more it is sweet, and, if, tested at once, sugar is found in abundance. This difference in time, in the activity of the agent, is in striking contrast with that of common organic matter in a state of change when acting on starch.

MM. Robin and Verdeil, from similar facts deny that pepsin is a special agent in the gastric juice; they regard it as organic matter in process of change, which, with an acidulated fluid, will, they assert, digest articles of animal food. A fluid of that composition will dissolve raw or cooked meat, but requires fifteen or twenty hours to effect it. Nor has it been shown that the special peptone or albuminose, the constant product and object of gastric digestion, resulted from the solution of this artificial digestive fluid. This imperfect observation can not be accepted in the face of the daily experience of healthy digestion accomplished in from three to four hours.

The conversion of starch as an aliment begins in the mouth during mastication, and if in small quantities, is there completed; but when the food is largely amylaceous, the greater portion arrives in the stomach, where the process is continued but not finished. The unchanged starch passing into the intestines, is brought under the action of pancreatin and the intestinal juice, and is rapidly transformed into glucose. When animals are fed exclusively on starch for some days, a portion is found in the fæces unchanged; the larger part has disappeared in the intestinal canal. (*Lehmann.*)

It has been denied that starch is ever changed into glucose in the stomach, or that it can take place in the presence of an acid. (*Fremy and Boutron.*) This fact has been decided as respects the human stomach, by the experiments of Grunewald and Schroeder, who had under their charge a woman with a gastric fistula produced by a wound. They were communicated to M. Longet by M. Shiff, who witnessed the experiment. "Some ounces of hydrated starch were introduced into the stomach through the fistulous opening while fasting. Immediately after some starch was expelled, which was found already to contain sugar. In a quarter of an hour sugar was found abundantly in the stomach, and the whole of the starch was fluid (dextrin)."*

M. Bidder asserts that the power of changing starch into sugar in the stomach persists in the presence of free acids; and M. Ernst Schroeder states that the saliva possesses its activity in the stomach, and rapidly transformed swelled starch (turgefactum amylum) into sugar.†

This error arose from making the experiment on dogs, in which starch is very imperfectly changed, if at all, by their saliva. Starch does not enter into the natural food of that animal, and it appears as though no provision was made by nature for an office not intended in the natural state. If this view be correct, it is confirmative of the theory that there is a special agent, diastase, in the salivary fluid. Longet expressly objects to the using of these animals in this investigation; he alleges that their saliva has a very low transforming power, and their gastric juice is very acid.

The experiments of Lehmann, Bernard, Longet, Corvisart, and others, have established the facts of the transforming power of the pancreatic and intestinal juices, and that dextrin and glucose are the common products into which all the amylaceous and saccharine substances in food are converted in the alimentary canal. Glucose must, then, be regarded as the *proper physiological sugar.* Bernard has proved its existence in the earliest stages of fœtal development, before the formation of the liver, after which it is chiefly developed in that organ. He detected it in the fluids of the amnios and the allantoid.‡ In seeking for the origin of this sugar, or the

*Longet, Traite de Physiologie, vol ii, p. 174. †Ib., *loc. cit.*

‡ Leçons de Physiologie, vol. i. Leçon, xxi.

glycogenic matter which produced it, he observed in the placentas of rabbits and guinea-pigs a whitish substance formed of a mass of cellules filled with sugar-forming substance. These last resembled in this respect the liver-cells of the adult animal. In pursuing this investigation, M. Bernard ascertained that similar epithelial or glandular cellules existed in the placentas of mammifers in the first periods of embryonic life, producing a sugar-forming substance. This anatomical element of the placenta, in some animals of this class, is mingled with the vascular portion of the organ, but in the ruminants it is separated in the form of epithelial layers on the amnioas. In this manner, as Bernard had previously asserted, the fact is established that the production of an amylaceous sugar-making matter is a function common to animals and vegetables. The provision of transitory structure for producing dextrin and glucose in the earliest stage of the embryon, proves its importance in the nutritive actions and the organizing processes. They appear to be constituent principles of the organizable matter from which organized structures are formed.* In extending his researches, he discovered the same kind of cellules containing sugar-making substance to be diffused in certain tissues in this stage of embryonic development. M. Cl. Bernard gives a full and very clear exposition of the experiments by which he ascertained the above fact. As these are not material in this discussion, I refer those who would wish to see the details to the original memoir, and limit myself to little more than a mere sketch of the results he obtained. 1. The tissue of the skin is infiltrated with the glycogenic matter, which is contained also in the cells of the epithelium, as demonstrated by the microscope. Obtained a decoction of the skin, it manifested its essential character by being changed into sugar by the action of strong acids and under the influence of animal or vegetable diastase. It possessed all the properties belonging to the glycogenic matter of the liver and placenta. It is found also in the corneous appendages of the skin, as hoofs, claws, etc. ; it disappears as they become hard and organized. 2. The glycogenic cellules are readily demonstrated in embryons on the whole of the mucous membranes of the alimentary canal, from the mouth and tongue to the end of the large intestines; they are seated in the epithelium which covers the villosities. They also exist in the glandular ducts

* Comptes Rendus de l'Acad. des Sci., vol. xlviii. pp. 77-86.

opening on the mucous membranes. 3. The same cellules are present in the mucous membrane of the bronchial tubes aud of the nostrils. These cellules disappear, existing only during a limited period of embryonic existence; notwithstanding, the sugar-making matter remains diffused through the other portions of the pulmonary structure, and remains persistent until birth, as is shown by means of a decoction of the lung, in which it can be detected. 4. In the genito-urinary mucous membranes the same cellules are present during their evolution; they were found in the uterus, Fallopian tubes, bladder, ureters and canaliculæ of the kidneys.

In the above instances the glycogenic cells and substance were observed existing the first stages of development. They are only temporary; and as soon as the permanent epithelia are formed, then they disappear.

5. The muscles in the earliest state of development are preceded by embryonic cells in which no sugar-making substance can be detected by the microscope or by chemical reactions. But at a later period, when the histological elements appear, the glycogenic matter is perceived interposed between the nuclei, and gives its characteristic color on the application of the reagents employed. When the muscular fibre has acquired its development, the glycogenic matter then appears to be infiltrated in the substance of the fibre.

The smooth muscles contain the sugar-making substance, like the striated; it can not, however, easily be detected by the microscope; but when a decoction is prepared from them, then it is found in great abundance, as it is when the striated are treated in the same manner.

The sugar-making substance is persistent in the muscles during the whole period of intra-uterine life, but disappears rapidly after birth.

It is a remarkable fact that the glycogenic matter in the fœtus does not exist in the nervous or osseous systems, or in the glands, appendages to the alimentary canal, as the salivary glands, pancreas, (except in the epithelia of their ducts,) and the glands of Lieberkuhn, the spleen, and lymphatic ganglions.

The liver does not manifest any signs of the presence of saccharine matters until the middle of uterine life. At this time it has acquired its histological structure, and then commence its functions by the formation of bile and the existence in it of glycogenic matter. As the liver enters into functional activity, the glycogenic cellules and matters disappear from the placenta, its envelopes, and the tissues that have been mentioned. Being a temporary organization and provisional function, at birth they have ceased to exist, and the liver enters on its permanent duties in the animal economy, lasting through life in its normal states.*

The preceding facts worked out by M. Cl. Bernard prove that in the first stage of the organic or nutritive actions, in animals as in vegetables, formative of plastic matter and organic forms, glucose is an indispensable agent. The organic, nutritive, and, as they may truly be named, vital actions, are identical in normal states through all the stages of existence, though, like all the functions, they are liable to be disturbed and perverted by various accidental influences. Yet the same law that presides over them in the embryonic state is persistent to the last span of life. It must be inferred from these facts that glucose is a physiological element in the vital or nutritive actions.

That nature, in milk, the food destined to nourish the young of the great class of mammalia, has made sugar a constituent is a significant fact pointing to the same conclusion. Sugar of milk, in chemical properties, belongs to the first species, or cane sugars, and is convertible into glucose. (*Lehmann.*)

The liver holds a prominent position in the physiological history of sugar. The immediate connection of this organ with the presence of sugar in the animal organism is the great discovery of M. Cl. Bernard. He established as a primary fact that the introduction of saccharine matters into the liver by the portal circulation was incidental and intermittent, while the presence of glycogenic matter was persistent, and sugar was always to be found in the blood of the hepatic veins issuing from the liver. The proportion constantly supplied to the organism in this way was 1 to 1.50. When it exceeds 3 of dried blood, it appears in the urine. These facts have been verified by all the prominent physiologists of Europe. Subsequently, M. Cl. Bernard completed this investigation by proving—first, that the glycogenic substance belonged to the liver; that the sugar may be completely washed out of that organ, removed from the body, and be again reproduced after a few hours; and second, by procuring it in a purified state, entirely isolated from the liver and all animal structure. Obtained in this state, it presented all the physical

*Comptes Rendus de l'Acad. des Sci., vol. lviii, pp. 673-684.

and chemical characters of starch and dextrin.* M. Biot tested it in his polariscope, and found a decided rotatory action to the right. An experiment of M. Pelouze is quite conclusive. He treated one gramme of this purified glycogene with fuming nitric acid, and obtained by the process xyloidine or gun-cotton, similar to that procured from vegetable starch. It was very combustible, and detonated at 180° C. It was converted also into oxalic acid, and by analysis gave a chemical composition that corresponded with the formula $C_{12}H_{11}O_{11}$. This glycogenic substance of the liver is transformed in the manner of starch into glucose, by an animal diastase existing in the liver and blood. There are numerous interesting facts connected with the glucogenic function of the liver, but which are omitted as irrelevent to my present object, which is to prove the importance of glucose and lactic acid in the organism as essential agents in organic or nutritive actions.

This view might have been taken from an *a priori* deduction. The immense amount of sugar and starch that enter into the food of man and animals, all of which are converted into glucose in the alimentary canal, are a strong indication of its necessity in sustaining vital activity. Dr. Stolle, some years since, estimated the cane sugars produced and consumed as food at 5,145 millions of pounds per annum.† Since then the cultivation of sugar has been extended, and the sorghum has been introduced into this country and Europe, the syrup of which is largely consumed. To this must be added the great amount of starch that forms so large a portion of vegetable food, together with fruits rich in glucose, as grapes, raisins, dates, figs, bananas, and other tropical productions, and the actual sum of glucose used up yearly in animal organisms will be found little short of double the above estimate. The extent of the cultivation, production, and consumption of the numerous substances for this one object, discloses an instinctive want essential to perfect animal existence, and to supply which nature has made ample provision. Although the exterior resources are so abundant, yet nature, in her foresight, has guarded against the many accidents that, in the eventful lives of man and animals, subject them to the total or partial want of vegetable food, has endowed the liver with its glucogenic function, by which the organism is sup-

* Comptes Rendus, vol. xliv. p. 578.

† Johnson's Chemistry of Common Life, vol. i. p. 224.

plied with glucose. In animals that die of starvation, this office is continued until about the third day before death. Life is protracted, and opportunity given for the intervention of new changes or more favorable events

Having shown the physiological position and importance of glucose in animal organisms, the next step is to follow it in its changes to its termination. A characteristic of glucose is its little stability, less than that of any other member of its group, which adapts it to its special offices in the economy. It disappears from the alimentary canal and the blood, as has been previously stated, while cane sugar, injected into a jugular vein, reappears unchanged in the urine.

In healthy conditions, glucose never accumulates in the blood or is found in any of the secretions, though it is introduced into the circulation without intermission from the liver or the alimentary canal, or both. It must, therefore, either be destroyed or transformed into some other constituent. In certain conditions, not properly ascertained, the reverse takes place, and the blood and whole organism are charged with glucose, and the urine is loaded with it, forming diabetes mellitus. What those conditions are has not been clearly determined. In fatal cases of yellow fever the liver possesses a light ochre color, and, from a limited number of examinations appears overloaded with an amyloid substance, with an entire absence of sugar. A somewhat similar state, but in less degree, it is said, occurs in fatal cases of bilious and intermittent fevers during the paroxysms. It would be an interesting inquiry to examine the liver in this point of view in all malignant forms of fever.

The conversion of cane sugars and amyloid matters of food into glucose is effected in the alimentary canal. In some animals, as dogs, this change does not take place in the stomach as proved by Bernard, but is promptly effected in the duodenum by the pancreatic fluid. In the human subject, hydrated starch is rapidly converted into dextrin and glucose, absorption of which takes place to a certain extent. That absorption of these products occurs, is rendered highly probable by the fact ascertained by Bernard, that cane sugar introduced largely into the stomachs of animals, is detected unchanged in the portal blood. A portion is also changed into lactic acid. This is admitted by both Lehmann and Bernard, who state that lactic acid is often formed in the stomach from glucose by the disturbing action arising from the digestion of the albuminous matter of the food.

Glucose is spread somewhat rapidly throughout the intestinal canal, being found in the cæcum often in an hour after its entrance. It exists as a thin, sometimes clear solution. It disappears in varying times, by two processes—conversion into lactic acid and by absorption. Diversities of opinion prevail on these points, but I follow chiefly Bernard and Lehmann as my authorities. After an abundant meal of saccharine or farinaceous substances the small intestines are decidedly acid; strongest in the duodenum, and feebler in the ileum. Lehmann asserts that this free acid "is lactic acid, according to direct experiments." Butyric acid is often found in the cæcum and colon. The absorption of glucose is rendered apparent from its rapid increase in the blood after a full meal of amylaceous or saccharine food (*Lehmann*).

The diffusive power of glucose is feeble, and its endosmotic equivalent low; hence its absorption is slow, and varies with the strength of its solution. This fact may explain why it is so seldom found in the portal blood, from the difficulty of detecting it in small quantities in that heterogeneous fluid. The whole of the glucose coming from the exterior, the representative of the amylaceous and saccharine food is disposed of in this way by conversion into lactic acid, and absorption into the circulating fluid.

The final destination of the interior supply of glucose, poured in a continuous stream from the heart into the general circulation in normal states of animal existence, can have but one solution. Glucose can not continue to exist under the influence of oxygen, heat and moisture, and in the midst of the unceasing conflicts of chemical atoms in the blood, and is transformed into lactic acid. Thus the final object of the vast provision of amylum and sugars in nature is to procure a constant supply of lactic acid, which thus appears indispensable in supporting vital activity in animal organisms.

This conclusion leads to a second inquiry, as to the uses of the lactic acid. Lehmann, in his *Manual of Chemical Physiology*, says "the physiological importance of lactic acid must not be too lightly esteemed."* It is so constantly present in the animal economy, that it may be looked on as one of its constituents. The subject has not been thoroughly handled. Liebig quotes the question, "What purposes does lactic acid serve in the organism?" and says it is of peculiar importance.† He does not solve it. His conclusion is that it

* Morris' translation, p. 78. † On the Chemistry of Food, p. 101.

is employed to support the respiratory process.* Lehmann and Bernard have gone more fully into the discussion. Their views are from different stand-points, and are, of course, dissimilar, but are not incompatible, and may be each adopted. Lehmann's view is purely chemical and physical, Bernard's is physiological. They are presented in the following brief summary:

1. The acid of the gastric juice has long been an unsettled physiological problem. Lehmann states "it (lactic acid) is the actual cause, with hydrochloric acid, of the digestive power of the gastric juice."† It is certain that no other acids can take their place in an artificial gastric juice. Bernard, from numerous experiments, concludes that lactic is the natural acid of the gastric juice; and Liebig, from Lehmann's own experiments, by correcting an error, decides, "in that case the gastric juice contains lactic acid."‡

2. In the intestinal canal, during digestion of mixed food, albumen and gelatin peptones, the products of the digestion of animal food, become acidulated by the free lactic acid with which they are mixed. According to Graham, acid bodies possess great diffusibility; and Jolly has proved that alkaline bodies have a low diffusive power, but are strongly osmotic. Hence the acidulous digested peptones separated from the alkaline blood in the capillaries of the mucous membrane are rapidly transferred into the circulation by the active endosmose excited in this manner.

3. The lactic acid, by the same physical properties and by the above process by which it promotes the introduction of nutritive materials into the organism, effects the elimination of effete and recremental matters out of the system. This action is most marked in the muscular system, which forms the chief mass of the higher animals. The muscles are strongly imbued with a special fluid (the muscular juice), contained in the interior of the fibrillæ. We are indebted to Liebig for a full knowledge of this fluid. It consists of the effete substances creatin, creatinin, and inosic acid, with alkaline salts held in solution by an acid fluid. Lactic and phosphoric acids, and acid lactates and phosphates, cause its acidity, of which lactic acid is the most important. The elements of active endosmose are here present: an alkaline albuminous fluid in the fine capillaries on one side, and an acidulous fluid in the other, sepa-

* On the Chemistry of Food, p. 103.

† Lehmann's Manual of Chemical Physiology, Morris' translation.

‡ Chemistry of Food, p. 138.

rated by little more than basement membrane must cause a constant flow of the muscular juice into the blood, in which the above effete matters are found to exist, which are rejected by the kidneys, and again met with in the urine, except the inosic acid, decomposed in the circulation. Liebig denies the constant presence of lactic acid or lactates in the urine. He relates an experiment made with "three persons who took a quantity of lactate of potash sufficient to have yielded an ounce of lactate of zinc. Before the experiment the urine had an acid reaction; immediately after it was alkaline, and the potash was detected in it, exceeding in quantity that in ordinary urine. The lactic acid could not be detected in it; it had disappeared in its passage through the blood." Lehmann concludes from* this ready combustibility of its alkaline salts that lactic acid becomes an important assistant in maintaining the animal temperature. This is true, but not more so than any other of the thousands of never-ceasing oxidations going on during life throughout the whole organism, each one contributing its mite, its minimum degree of heat. By this method nature secures the remarkable equilibrium of animal heat indispensable to health and existence.

4. The views of M. Cl. Bernard are almost entirely physiological. He lays down a proposition, or it may be termed a law, which, it appears to me, must be admitted as the expression of an important truth. It is, that whenever vital phenomena are manifested in acts of organization, two circumstances are to be kept in view: the first is the being, form or tissue in a state or process of development; and the next is the organizable matter or medium in and from which the development is taking place. The first will be passed by, as not of moment at this time, and I proceed to the second, in which will be found the examples of the principles it is attempted to establish.

Organic cells or tissues, when in the act of nutrition or development, must call incessantly on the medium of organizable matter that surrounds or bathes them for the materials of their organic composition. Two conditions are required in the organizable matter to adapt it for that purpose: instability of its chemical combinations, and an extreme mobility of the organic molecules, the preordained arrangement of which gives origin to specific cells and tissues. These are the primary and essential phenomena of life, and enable the organic chemical elements to be grouped and disposed of uninterruptedly in

*On the Chemistry of Food. J. Liebig. p. 102.

thousands of modes under germ action. Animal and vegetable cells and tissues can be developed only from a plastic matter in which the organic chemical elements and the organic molecules are prevented from falling into a fixed or statical condition, in which consists the chemical and molecular stability, the characteristic of dead and inorganic matter. Dry gangrene and local embolism may be cited as examples.

This unceasing play of the chemical elements and of molecular activity, so essential to all the vital phenomena of germ development, assimilation, nutrition, secretion, etc., are effected by chemical and physical causes and agents coming from the exterior. Of these the most active and powerful is oxygen; next in power and energy is lactic acid, not glucose. The mere conversion, new arrangement of its chemical atoms, could give no impulse to molecular and chemical disturbance in other bodies, even in contact. But lactic acid, as soon as formed, seizes on the alkalies, converts them into lactates, "which are destroyed as fast as they are produced." (*Liebig.*) In this instance we are presented with the extraordinary phenomenon, persistent through life, of the production of a complex chemical body in the blood, and its almost immediate destruction, apparently without a sufficient object. The lactates have feeble resisting power. Oxygen, at the temperature of the body, and in its active state (ozone), tears asunder the chemical atoms of the acid, forming carbonic acid and water, its oxygen is set loose in a nascent state (highest intensity of chemical force), and the alkalies escape through the kidneys. The blood, by these combined agents, oxygen and lactic acid, is the seat of intense molecular commotion. This state of the organizable matter, it has been shown, is a *sine qua non* of all vital actions. The enigma above stated is solved. The final result of this discussion is that the intention of the immense provision of amylaceous and saccharine substances in the food of animals is the constant production of lactic acid in the blood of animals, to assist in the support of vital motion in the blood. The mode of action of some deadly poisons bears comfirmative evidence in favor of the doctrine. Carbonic oxide, hydrocyanic acid, sulphuretted hydrogen, chloroform appear to exert their fatal effects by arresting the chemical action of oxygen in the blood. Carbonic oxide expels the oxygen of the circulating fluid, though it retains the arterial hue even in the veins. (*Bernard.*) The blood, in poisoning by hydrocyanic acid, is of a dark hue, neither venous nor arterial; it is the same for sulphuretted hydrogen. Exposed to the air or oxygen, the color is not changed. The same facts attend the poisoning by aconitia and veratria. The blood has been reduced to a quiescent state—to a statical condition, and sudden death is the inevitable consequence. Their mode of action on external bodies shows the correctness of our explanation. They prevent fermentation, and arrest it when in progress. Now Pasteur has demonstrated that fermentation is a vital and not a chemical phenomenon, produced by the development and nutritive action of living organisms, vegetable and animal. In alcoholic fermentation sugar is decomposed by the torula cerevisia, which abstracts a part of its carbon and hydrogen for its own nutrition. The remaining constituents enter into new combinations forming vegetable carbonic acid and alcohol. The vitality of the organism is destroyed by the poison, and the fermentation ceases. Modern physicists have demonstrated that light, heat, magnetism, and electricity, the great forces of nature, are varieties of motion. This investigation has led to a similar conclusion, that all purely organic and vital phenomena arise from atomic disturbance and molecular motion.

In the preceding discussion I have adhered to the doctrine of M. Cl. Bernard that the heart injects without intermission into the blood, in normal conditions, a certain quantity of glucose. I am perfectly aware that the fact has been contested by Dr. F. W. Pavey. I have not been convinced by his facts or his argument. He starts with the assumption that all the examinations of the blood, before his own, had been made after death, and that no one had taken the blood from the right heart by catheterism except himself. In this statement he was certainly mistaken. M. Cl. Bernard, in his physiological winter course of 1855, on the 9th of January, in his fifth lecture, made a comparative experiment on two dogs, the one fasting, the other in the act of digestion after a full meal of animal food. The object of the experiment was to show "the physiological oscillations in the distribution and amount of sugar in the blood under the influence of digestion." This was done by taking portions of blood, from the right ventricle, the carotid, and the jugular of each animal. The blood was obtained from the right heart by catheterism—"*en pratiquant une sorte de catheterisme cardiaque*"—which was performed by a simple instrument, of which he gives a figure, and he designed a simple and neat method of operating which he describes. It is omitted for want of space. The results were, that of the samples taken from the first dog (fasting) in that from the right ventricle alone was sugar detected in small amount; but those from the second dog all yielded sugar, that from the right ventricle quite abundantly, less in that from the carotid, and still less from the jugular.* These conclusive experiments appear to have been unknown to Dr. Pavey.

M. Chaveau, 1856, presented to the Academie des Sciences a series of experiments on the blood of large quadrupeds, as horses, asses, mules, etc. His connection with the Imperial Veterinary School of Lyons gives him great facilities for experimenting with animals. He collected specimens of blood from horses and dogs fasting from twelve hours to six days. The blood was obtained from the coxigeal, femoral, and carotid arteries, and their collateral veins, and from the heart. A quantitative analysis was made with a uniform result; sugar was found in all the different portions of blood, that of the arteries giving the highest figures, and that of the veins the lowest. The blood from the two hearts was nearly the same. But the capital and decided fact was the catheterism of the sub-hepatic veins through the jugular, after the ligation of that vessel, and the auricle into the posterior vena cava; and the blood obtained was the richest in sugar. M. Chaveau remarks that his method was entirely physiological and not open to objection, for in operating on solipedes the animal was always standing (debout†).

I wish to state, in conclusion, that my young friend, Dr. W. F. Atlee, consulting surgeon to the U. S. Satterlee Hospital, two years since treated gangrenous wounds successfully with sugar. This discovery has been confirmed by Dr. J. H. Packard, one of the surgeons to U. S. Hospital, Beverly, N. J.‡ The active agent in this treatment is not the sugar, as is supposed, but lactic acid, into which "sugar of milk, starch, grape sugar, and cane sugar are converted by *contact* with animal substances in a state of decomposition." (*Liebig.*) I would suggest to the gentlemen, surgeons of the U. S. hospitals, in which this formidable affection has frequently prevailed, that they should give a trial of the effects of a direct application of lactic acid. It can be done very readily by using very sour milk. The wound ought to be thoroughly washed with milk, and cleansed of putrid matter, and then covered with pledgets saturated with the acid fluid frequently renewed.

*Leçons de Physiologie Experimentale, vol. i, leçon v, pp. 119–122.

†Comptes Rendus de l'Académie des Sciences, vol. xiii, pp. 1008–12.

‡See January No. of this Journal.

This explanation is supported by the well known fact, that the gastric juice, in which lactic acid is always present, is antiseptic, and arrests putrefaction.—*Am. Jour. of Med. Sci.*

